# Diabetes in Pregnancy and Risk of Antepartum Depression: A Systematic Review and Meta-Analysis of Cohort Studies

**DOI:** 10.3390/ijerph17113767

**Published:** 2020-05-26

**Authors:** Kai Wei Lee, Siew Mooi Ching, Navin Kumar Devaraj, Seng Choi Chong, Sook Yee Lim, Hong Chuan Loh, Habibah Abdul Hamid

**Affiliations:** 1 Department of Family Medicine, Faculty of Medicine and Health Sciences, Universiti Putra Malaysia, Serdang, Selangor 43400, Malaysia; lee_kai_wei@yahoo.com (K.W.L.); knavin@upm.edu.my (N.K.D.); 2 Malaysian Research Institute on Ageing, Universiti Putra Malaysia, Serdang, Selangor 43400, Malaysia; 3 Department of Psychiatry, Faculty of Medicine and Health Sciences, Universiti Putra Malaysia, Serdang, Selangor 43400, Malaysia; sengchoi@upm.edu.my; 4 Department of Nutrition and Dietetics, Faculty of Medicine and Health Sciences, Universiti Putra Malaysia, Serdang, Selangor 43400, Malaysia; l.sookyee@yahoo.com; 5 Clinical Research Centre, Hospital Seberang Jaya, Ministry of Health Malaysia, Perai 13700, Pulau Pinang, Malaysia; lohhongchuan@gmail.com; 6 Department of Obstetrics and Gynaecology, Faculty of Medicine and Health Sciences, Universiti Putra Malaysia, Serdang, Selangor 43400, Malaysia; habib@upm.edu.my

**Keywords:** antepartum depression, gestational diabetes, pre-existing diabetes, diabetes in pregnancy

## Abstract

Previous literature has reported that patients with diabetes in pregnancy (DIP) are at risk of developing antepartum depression but the results have been inconsistent in cohort studies. We conducted a systematic review and performed a meta-analysis to quantify the association between DIP and risk of antepartum depression in cohort studies. Medline, Cinahl, and PubMed databases were searched for studies investigating DIP involving pregnant women with pre-existing diabetes and gestational diabetes mellitus and their risk of antepartum depression that were published in journals from inception to 27 December 2019. We derived the summary estimates using a random-effects model and reported the findings as pooled relative risks (RR) and confidence interval (CI). Publication bias was assessed using a funnel plot and was quantified by Egger and Begg’s tests. Ten studies, involving 71,036 pregnant women were included in this meta-analysis. The pooled RR to develop antepartum depression was (RR = 1.430, 95% CI: 1.251–1.636) among women with gestational diabetes mellitus. Combining pregnant women with pre-existing diabetes mellitus and gestational diabetes mellitus, they had a significant increased risk of developing antepartum depression (RR = 1.431, 95% CI: 1.205–1.699) compared with those without it. In comparison, we found no association between pre-existing diabetes mellitus in pregnancy (RR = 1.300, 95% CI: 0.736–2.297) and the risk of developing antepartum depression. This study has a few limitations: first, different questionnaire and cut-off points were used in evaluation of depression across the studies. Second, there was a lack of data on history of depression prior to pregnancy, which lead to confounding bias that could not be solved by this meta-analysis. Third, data were dominated by studies in Western countries; this is due to the studies from Eastern countries failing to meet our inclusion criteria for statistical analysis. Women with gestational diabetes mellitus have an increased risk of developing antepartum depression compared to those without the disease. Therefore, more attention on the mental health status should be given on pregnant women diagnosed with pre-existing diabetes mellitus and gestational diabetes mellitus.

## 1. Background

Depression is a common illness worldwide [1,2] and women are about twice as likely than men to develop depression during their lifetime [3]. Without doubt, pregnancy is a major life event that is usually accompanied by hormonal changes and it is a time of extreme increased vulnerability for having antepartum depression [4,5]. Studies have reported that the prevalence of antepartum depression was higher in the second and third trimesters (12.0%–12.8%) as compared with the first trimester (7.4%) [6,7,8].

Based on the existing systematic review conducted among the Indian women, Arora and Aeri in 2019 summarized that the significant risk factors for antenatal depression are unplanned pregnancy, being a multigravida, having a history of abortion, advancing age, lower socio-economic status, lower educational status, unemployment, bad relationship with her in-laws, male gender preference, and excessive demand for dowry [9]. However, diabetes in pregnancy (DIP) was not included in that systematic review. In fact, there has been literature reporting data on the occurrence of antepartum depression among women with and without DIP [10,11,12,13,14,15,16,17,18,19,20,21,22,23,24,25,26,27,28]. Furthermore, DIP could be associated with the onset of antepartum depression.

On the other hand, most of the existing literature focused on the association between DIP and postpartum depression, for example a meta-analysis by Arafa and Dong [29] that reported that GDM is a significant risk factor for postpartum depression (pooled relative risk (RR) = 1.32). Another meta-analysis by Azami et al. found a similar pooled RR of 1.59 based on observational studies [30]. Indeed, postpartum depression has a drawn out ramifications for women and their children [31]. Nevertheless, studies have reported that the prevalence of antepartum depression (that ranged from 6.5% to 12.9%) [32] and postpartum depression (that ranged from 6.6% to 8.5%) were actually comparable to each other [33]. In fact, antepartum depression was also associated with postpartum depression [34].

Women with GDM have an increased risk of developing type 2 diabetes (with a RR of 7.43) compared to pregnant women who are normo-glycemic [35]. As it is well known, pregnant mothers with pre-existing diabetes mellitus (DM) are at a high risk for adverse pregnancy outcomes such as unplanned caesarean section, abnormal fetal birth weight, and congenital anomalies in the offspring [36,37]. Therefore, DIP (pre-existing DM and GDM) is known to be associated with increased risk of maternal and neonatal morbidity and mortality [7,38]. In mothers facing the dual challenge of pregnancy and upcoming motherhood, the association of DIP and depression imposes important health concerns. Currently, the relationship between these conditions remains indistinct due to the reason that the existing systematic review on the association between depression and DIP included all observational studies, whereby the causal relationship between DIP and depression was difficult to determine from the study design itself. Furthermore, some of the studies had the diagnosis of depression confirmed in the study’s participants even before the diagnosis of diabetes was made [39]. Therefore, we conducted a systematic review with meta-analysis to determine the association between DIP and the risk of antepartum depression only among the available cohort studies.

## 2. Methods

The present study was registered with the National Medical Research Register, Ministry of Health Malaysia (registration number: NMRR-20-674-53879). We followed the Preferred Reporting Items for Systematic Reviews and Meta-Analyses (PRISMA) criteria when conducting this meta-analysis and reporting its results [40].

### 2.1. Literature Search

Two investigators (K.W.L. and S.C.C.) independently searched Medline, Cinahl, and PubMed databases for potential studies published in journals from inception to 27 December 2019. We considered any relevant studies in the search as long as it was published before or on 27 December 2019. We also did not impose a limitation on the years of publication on the studies identified from reverse–forward citation tracking. We used a combination of search terms: (mood disorder OR unipolar depress* OR depress* OR depress* disorder OR major depress* OR major depress* disorder OR atypical depress* OR melancholi* OR melancholi* depress* OR melancholi* feature OR peripartum depress* OR persistent depress* disorder OR dysthymic disorder OR dysthymi*) AND (gestational diabetes OR diabetic pregnancy OR diabetes mellitus OR type 1 diabetes mellitus OR type 2 diabetes mellitus OR NIDDM OR non-insulin dependent diabetes mellitus OR insulin dependent diabetes OR pregnancy diabetes mellitus) for related studies. The search strategies are shown in Table S1.

### 2.2. Study Selection

Firstly, relevant articles identified through the databases were imported into Endnote program X5 version and any duplicate publications were removed. This step was performed by two investigators (K.W.L. and S.Y.L.) independently. Secondly, two investigators (K.W.L. and S.Y.L.) independently screened the titles and abstracts of those articles for suitability based on the search strategies mentioned above. Thirdly, full-text articles were assessed based on the inclusion criteria mentioned below by two investigators (K.W.L. and S.Y.L.) independently. Any disagreements were resolved by discussion before commencing the quantitative analysis. In addition, we manually performed reverse–forward citation tracking of the identified studies. This step was also performed by two investigators (K.W.L. and S.Y.L.) independently.

### 2.3. Inclusion Criteria

Cohort studies were eligible for quantitative analysis if the study’s participants consisted of those with and without DIP. The studies were also required to present data of antepartum depression screened or diagnosed at either the second or third trimester as a primary or secondary outcome. The studies must have been published in an English peer-reviewed journal. Studies were excluded if the samples size was less than 100 or there was no information on which trimester the depression assessment was conducted. We also excluded studies that did not show any data relevant to a correlation between DIP and antepartum depression; these studies were categorized as “insufficient data” in PRISMA flowchart.

### 2.4. Data Extraction

The following data were extracted by two reviewers (K.W.L. and S.Y.L.): the last name of the first author, year of publication, country, ethnic origin, mean age or median of participants, number of participants with diagnosis in pregnancy among those with or without depression symptoms, study tool for assessment of depression, cut-off value for diagnosis of depression, and trimester where depression assessment was done. Data extraction was conducted independently, and the results of data extraction were compared between the two reviewers to ensure no errors.

### 2.5. Exposure and Outcomes Measures

Data regarding exposure to pre-existing DM are referred as pre-gestational diabetes (type 1 or type 2 DM diagnosed before pregnancy) [41]. GDM is defined as glucose intolerance of variable degree with onset or first recognition during pregnancy [42]. Diabetes in pregnancy (DIP) could refer to pre-existing DM and/or GDM.

Measures of exposure for DIP were derived in three ways. First, the number of DIPs was calculated by summing the number of pregnant women with pre-existing DM and GDM if both data were available separately in original articles. Second, if pre-existing DM and GDM were presented as diabetic pregnant women in original articles in which differentiating the data between pre-existing DM and GDM could never be possible, we used the number of diabetic pregnant women in calculation of the number of DIPs. Third, if the articles indicated the numbers of pregnant women with either pre-existing DM and GDM, the data of available groups (either pre-existing DM or GDM) were used in calculation for the numbers of DIPs and its risk for antepartum depression.

The outcome was the presence of antepartum depression, where it could be determined either with a confirmed diagnosis or use of screening tools. Any diagnostic guidelines or screening cut-off value for depression was acceptable for data synthesis in the meta-analysis. Measures of outcomes were the number of pregnant women with antepartum depression.

### 2.6. Data Synthesis

We used the relative risk (RR) and the corresponding 95% confidence interval (CI) to quantify the association between DIP and depression for observational studies. The meta-analyses were performed using a random-effects model of DerSimonian and Laird, which incorporates both within and between-study variability, as we anticipated between-study heterogeneity. Heterogeneity across studies was assessed using the I^2^ index (low was <25%, moderate 25%–50%, and high >50%), that indicated the total per cent of discrepancy due to studies variation [43].

### 2.7. Quality Assessment

The quality of the individual studies was determined using the checklist Strengthening the Reporting of Observational Studies in Epidemiology (STROBE) [44]. Two investigators (H.A.H. and N.K.D.) individually assessed the studies quality, and any discrepancies were resolved by discussion with the third investigator (S.M.C.). Studies were nevertheless included in analysis regardless of the STROBE score and grading.

### 2.8. Statistical Analysis

A random-effects (DerSimonian and Laird Method) meta-analysis was used throughout the analysis to compute the pooled RRs and their 95% CI [45,46]. We also examine potential publication bias by funnel plot, Begg’s test, and Egger’s test [47], and excluded those studies with high risk of publication bias from meta-analysis, which might be the source of heterogeneity. Heterogeneity across studies was assessed using the I^2^ index (low was <25%, moderate 25%–50%, and high >50%) [46]. A sensitivity analysis was conducted using leave-one-out meta-analysis to examine how each individual study affects the overall estimate of the rest of the studies. All analyses were performed using Open Meta(Analyst) software, this software can be accessed and downloaded from http://www.cebm.brown.edu/openmeta/index.html [48].

## 3. Results

### 3.1. Description of Included Studies

**Figure 1:**
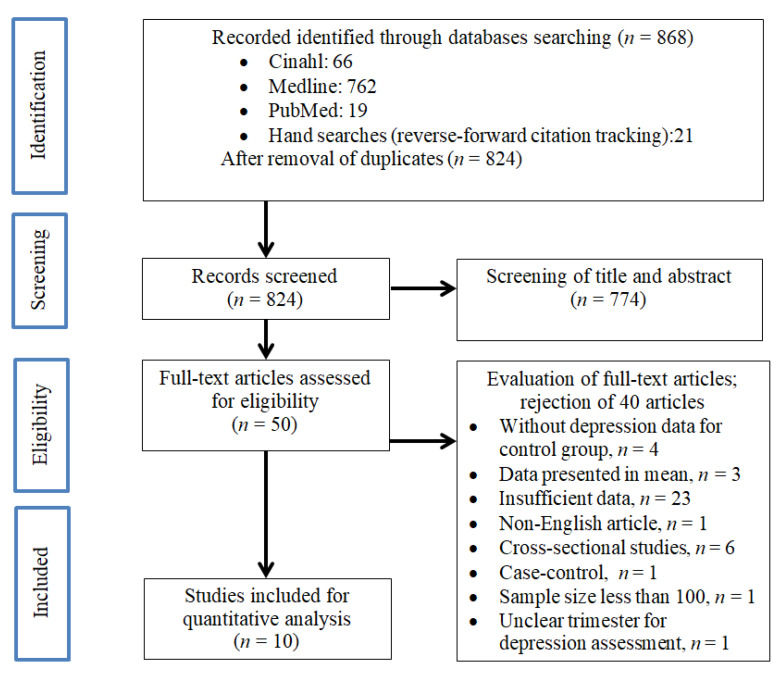
Preferred Reporting Items for Systematic Reviews and Meta-Analyses (PRIMSA) flow diagram of the literature screening process.

Our literature search identified 868 articles in the initial screening as shown in Figure 1. After removal of duplicate articles (*n* = 44), a total of 824 studies were retrieved for review of title and abstract. After screening for its suitability through title and abstract, 50 studies were subjected to full-text assessment for inclusion criteria. After careful evaluation of the 50 articles, 10 studies were eligible for quantitative analysis in this study.

### 3.2. Characteristics of Included Studies

The characteristics of the included studies are summarized in Table 1. A total sample of 71,036 pregnant women were included in the analyses. Among the 10 studies, six studies were conducted in USA [15,18,20,22], two studies were conducted in Australia [16,26], and one study conducted each in Brazil [11], Canada [24], Greece [27] and Ireland [17], respectively.

In terms of screening for depression, Edinburg Postnatal Depression Score was the most commonly used assessment tool [18,26,27]. In addition, different cut-off points at 12 and 13 were used in Edinburgh Postnatal Depression Score, which differed across three studies. Other screening tools such as the Depression Anxiety Stress Scale [17], Mental Health Inventory Form [16], and Primary care evaluation of Mental disorders [11] were used in some of the studies. For diagnostic purposes, the International Classification of Disease [20,24] and Patient Health Questionnaire [22] were used. There were also studies [15] in which the screening tool for depression was not mentioned.

Half of the included studies provided data of respondent ethnicity composition. As we could see that most of the respondents comprised of Caucasians. The mean age range was within 29 to 30.2 years old, however these values merely came from two studies that reported this value [11,26], and majority of the studies did not report mean age of respondent. In regard to quality of studies, the majority of studies had a score of 14 or above [15,16,17,18,20,22,24] except for three studies [11,26,27].

### 3.3. DIP and Risk of Antepartum Depression

**Figure 2:**
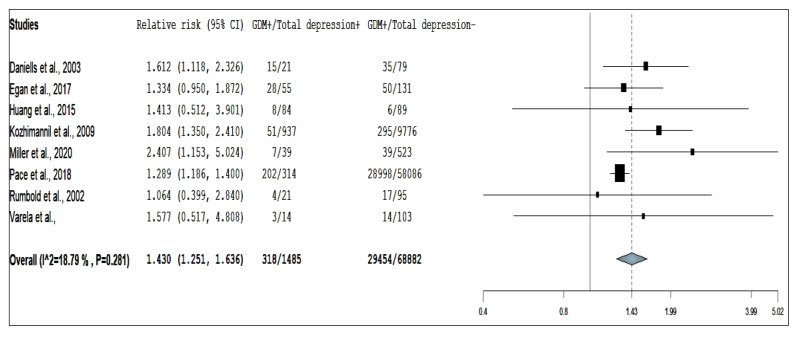
Forest plot of gestational diabetes mellitus and risk of antepartum depression.

**Figure 3:**
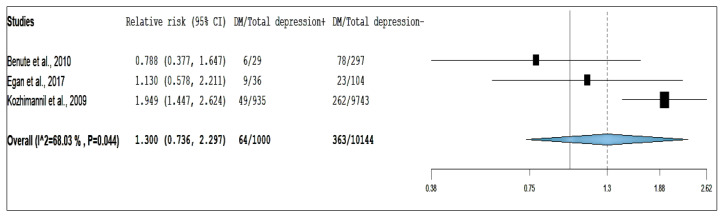
Forest plot of pre-existing diabetes mellitus and risk of antepartum depression.

**Figure 4:**
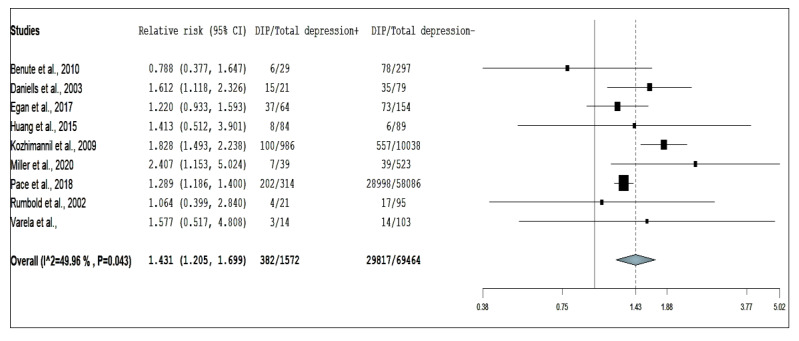
Forest plot of diabetes in pregnancy and risk of antepartum depression.

Eight studies reporting on the association between risk of antepartum depression and GDM, three studies for pre-existing DM, and nine studies for DIP were included for the overall analysis. The pooled RR using random-effect models are presented in Figure 2, Figure 3 and Figure 4.

The results suggest that women with GDM have a statistically significant 43% increase in risk of developing antepartum depression (pooled RR = 1.430, 95% CI: 1.251–1.636; Figure 2). There was low degree of heterogeneity across the included studies (I^2^ = 18.8, *p* = 0.281). The funnel plot, Egger’s test (*p* = 0.882) and Begg’s test (*p* = 0.621) suggested that there was no publication bias (Figure S1 and Table S3). Nevertheless, sensitivity analysis identified all studies had substantial influences on the overall relative risk, which cause variation in pooled RRs ranging from 1.313 to 1.605 [16,17,18,20,22,24,26,27].

Our study showed that pregnant women with pre-existing DM, compared with those without diabetes, had an insignificant pooled RR of 1.300 (95% CI: 0.736–2.297) and I^2^ for heterogeneity was 68.0% (*p* = 0.044; Figure 3). The test for the small-study effect suggests that there was evidence present based on Begg’s test (*p* Value= 0.117) but not on funnel plots and Egger’s test (*p* Value = 0.216; Figure S2 and Table S4) after the study by Cripe et al. [15] was excluded from analysis. The study by Cripe et al. [15] was removed from the analysis because it caused a high degree of heterogeneity (I^2^ = 92.3%, *p* < 0001) and the test for the small-study effect suggested that this study [15] may have publication bias, of which there was evidence present based on funnel plot and Egger’s test (*p* = 0.076) but not in Begg’s test (*p* = 1.000). Sensitivity analysis of all studies had substantial influences on the overall relative risk, which cause variation in pooled RRs ranging from 1.300 to 6.778 [11,15,17,20].

The association between DIP and antepartum depression was significant compared with those without DIPs (pooled RR = 1.601, 95% CI: 1.190–2.153, I^2^ = 82.5, *p* <0.001 for heterogeneity). Funnel plot and Egger’s test (*p* = 0.091) but not Begg’s test (*p* = 0.655) indicated that the study by Cripe et al. [15] may have had publication bias. Overall, all studies [11,15,16,17,18,20,22,24,26,27] affected the pooled RRs, causing it to vary from 1.431 to 1.779 after combining the number of pregnant women with pre-existing DM and GDM compared with those without diabetes. Due to the high heterogeneity and publication bias, we removed the study by Cripe et al. [15] from the meta-analysis. After the study by Cripe et al. [15] was excluded from analysis (Figure 4), the pooled RR was 1.431 (95% CI: 1.205–1.699) and the I^2^ reduced to 50.0% (*p* = 0.043). We also did not find any evidence of publication bias when we reassessed the funnel plot, Egger’s (*p* = 0.462) and Begg’s test (*p* = 0.532; for publication bias, both *p* > 0.20; Figure S3 and Table S5) after performing this crucial step [15].

### 3.4. Sensitivity and Publication Bias Analysis

Sensitivity analysis revealed that excluding any of the studies could cause significant changes to the pooled relative risk. The decision whether to exclude any study from the meta-analyses was made after considering the publication bias analysis as well as the heterogeneity prior to excluding any of the eligible studies. Based on the funnel plot, Egger’s test and Begg’s test, there was no evidence of publication bias in the meta-analysis of GDM. Therefore, we did not exclude any studies from the meta-analysis.

On the other hand, we found that the study by Cripe et al. [15] may have contributed to publication bias in meta-analysis for pre-existing DM and DIP and risk of antepartum depression. After removing the study by Cripe et al. [15] from the meta-analysis for pre-existing DM and risk of antepartum depression, the pooled RRs were reduced from 3.433 (95% CI: 0.988–11.927) to a RR of 1.300 (95% CI: 0.736–2.297) and heterogeneity was also greatly reduced from 92.3% to 68.0%.

Similar observation was also seen in the association between DIP and the risk of antepartum depression, in which the pooled RRs also declined (from pooled RR = 1.601 to pooled RR = 1.431 by excluding the study by Cripe et al. [15] and heterogeneity was reduced by nearly half from I^2^ = 82.5% to I^2^ = 50.0, which once again proved that the study by Cripe et al. [15] could have contributed to the publication bias (as shown by the funnel plot and Egger’ test, *p* = 0.091).

## 4. Discussion

To our knowledge, this is the first meta-analysis to comprehensively summarize the association between DIP and the risk of antepartum depression. This study aimed to assess whether pregnant women with pre-existing DM or GDM are indeed at a higher risk of developing antepartum depression.

In the present meta-analysis, it was demonstrated that the presence of GDM correlated with a 43% increased risk of having antepartum depression, and DIP presence also indicated a similar result in its association with antepartum depression. Even though this meta-analysis showed significant increased risk of antepartum depression in women with GDM, it would be too premature to say that DIP and antepartum depression have a strong association due to the individual biases of the studies and limitation of this study. Therefore, more studies are needed to look into this association. Having said that, a possible physiologic mechanism for this significant association could be linked to the secretion of cortisol and expression of certain inflammation markers in pregnancy [49], that are in turn associated with hyperglycemia and insulin resistance [50]. Therefore, the abnormal secretion of these stress hormones could be exacerbated in the presence of hyperglycemia and insulin resistance in pregnancy [50], which might lead to a heightened inflammatory response that is common among those with depression [49]. Another psychological explanation could be linked indirectly to fears and worries of the developing obstetrics complications among women with GDM, especially about the possible consequences for their unborn child and also resultant poor maternal health and distress that could lead to maternal depression [16,51,52].

Surprisingly, our study found a no association between pre-existing DM in pregnancy and the risk of developing antepartum depression. The statistical power could be limited by the mere inclusion of three studies having high heterogeneity for the current meta-analysis. Nevertheless, the lack of association could also be explained by the possibility that women with pre-existing DM were more aware of the importance of physical activity and weight control through various psycho-education or lifestyle modification programs they were exposed to prior to conceiving. This may also help in improving the insulin resistance and preventing the emergence of depressive symptoms [53]. In addition, individuals having a longer duration of diabetes could have better illness perception, and are therefore more able to cope constructively with their disease [54,55]. These facilitate self-efficacious behaviors among pregnant women with pre-existing DM [56], leading to better health outcomes and less depressive symptoms during pregnancy. However, the information on the duration of diabetes illness and the extent of anti-diabetic medication adherence were not available in these included studies. Thus, it was not possible to consider these variables in sub-group analysis.

A prior meta-analysis estimated that GDM significantly increased the risk of postpartum depression by a pooled RR ranging from 1.32 to 1.59 [29,30]. Meanwhile, we estimated that the association between GDM and risk of antepartum depression was statistically significant at with a pooled RR at 1.43. Even though antepartum and postpartum are two distinctive periods, there are similarities in finding of GDM and its risk on antepartum and postpartum depression. It can therefore be assumed that presence of perinatal depression could possibly be related to GDM. However, a prior systematic review and meta-analysis that reported the pooled prevalence [6,8,32] and the risk factors for antepartum depression [9] did not include GDM in the meta-analyses. Therefore, if new study is available in the future, a more detailed conclusion can be drawn. Until then, based on the available studies, this is the first study that shows a possible association between DIP and antepartum depression.

### 4.1. Implications of This Study

The co-existence of DIP and depression could be a lethal combination. Therefore, it is important to be able to identify pregnant women with diabetes at risk of developing antepartum depression earlier for various reasons. For the mother, antepartum depression is known to cause detrimental effects such as poor self-care, impaired quality of life, increased risk of suicide, and postnatal depression [57]. For the new-born babies, early recognition and intervention on antepartum depression can prevent the associated adverse perinatal outcomes such as preterm delivery, low birth weight, growth retardation, infants having diarrheal diseases or with disrupted cardiorespiratory regulation and feeding problem, as well as long term cognitive and behavioral deficits [58,59].

For healthcare providers, this indicates the need to screen for depression in woman with DIP. For policymakers, the latest clinical practice guidelines should clearly outline the need for those providing antepartum services to do this important screening.

In view of the fact that antepartum depression is a negative psychological effect of DIP, and both conditions are associated with an increased possibility of both maternal and neonatal adverse outcome [60,61], it is therefore pertinent to acknowledge DIP as one of the associated factor for antepartum depression.

### 4.2. Strength and Limitations

This is the first systematic review and meta-analysis that reported a significant association between GDM, DIP, and antepartum depression, in which it estimated the relative risk based on 10 cohort studies comprising of more than 71,000 pregnant women.

Our meta-analysis has a number of potential limitations. First, the questionnaire used in evaluation of depression varied across studies. Furthermore, we notice that different cut-off points at 12 and 13 were used in the Edinburgh Postnatal Depression Score, which differed across three studies. In a one of the studies the questionnaire used for assessment of depression was not even mentioned, which may have increased the risk of information bias. Second, data on the history of depression prior to pregnancy have been particularly helpful for subgroup analysis. However, the availability of these data was very limited leading to an increased risk of confounding bias that could not be solved by this meta-analysis. Third, data of the current study were dominated by studies in Western countries, and less so from Asian and African countries. Therefore, the finding of this study should be interpreted with caution. In fact, there are studies on the correlation between DIP and antepartum depression that have been reported from Eastern countries, but they were conducted with a cross-sectional design. We choose cohort study design as one of our inclusion criteria because it demonstrates a more causal relationship than cross-sectional study design. Therefore, many studies from Eastern countries were not eligible for statistical analysis.

## 5. Conclusions

In summary, our study suggested that the presence of GDM significantly increased the risk of developing antepartum depression. Given the appreciable relative risk of antepartum depression among those with DIP, more attention on the mental health status should be given to pregnant women diagnosed with pre-existing DM and GDM. Furthermore, more studies from Eastern countries with a well-designed prospective study design are needed to confirm our study findings.

## Figures and Tables

**Table 1:** Characteristics of the included studies in meta-analysis which comprising 73,845 pregnant women whom depression was assessed in second or third trimester.

Author, Year	Country	Ethnic Origin	Mean Age ± SD; Median (Range)	Study Tool for Assessment of Depression (Depression Type)	Cut Point for Diagnosis of Depression	Enrolment Trimester for Depression Assessment ^†^	Study Conclusion	Quality *
Benute et al., 2010 [11]	Brazil	N/A	30.2 ± 7.1	Primary care evaluation of mental disorders (major depressive disorder)	Presence of 4–6 depressive symptoms	28.2 week’s gestation ± 10.5	Unplanned pregnancy in women with a medical disorder was identified as a risk factor for major depression during gestation. Major depression during pregnancy in women with a medical disorder should be routinely investigated using specific methods.	Poor
Cripe et al., 2011 [15]	USA	N/A	N/A	Assessment tool was not mentioned (depression)	N/A	<20 week’s gestation	Pregnant women with a history of migraine may benefit from screening for depression during prenatal care and vigilant monitoring, especially for women with co-morbid mood and migraine disorders.	Good
Daniells et al., 2003 [16]	Australia	N/A	N/A	Mental Health Inventory form—5 items (major depression)	>16	30 week’s gestation)	There were no sustained increased levels of anxiety for women diagnosed with Gestational diabetes mellitus (GDM). Concerns expressed about causing sustained maternal anxiety by testing for GDM could not be substantiated.	Good
Egan et al., 2017 [17]	Ireland	Type 1 diabetes mellitus group (Caucasian, 96.9%; non-Caucasian, 3.1%); GDM group (Caucasian, 89.7%; non-Caucasian, 10.3%); Control group (Caucasian, 98.1%; non-Caucasian, 1.9%)	N/A	Depression anxiety stress scale—21 items (clinically significant depression: moderate–extremely severe)	≥14	Third trimester	This work highlights a potential role for targeted psychological interventions to address and relieve symptoms of anxiety and depression among pregnant women with diabetes.	Good
Huang et al., 2015 [18]	USA	White, African American, Asian and others	N/A	Edinburgh Postnatal Depression Score—10 items (depressive symptoms)	≥13	≤22 week’s gestation)	Pregnancy hyperglycaemia was cross-sectionally associated with higher risk of prenatal depressive symptoms, but not with postpartum depressive symptoms.	Good
Kozhimannil et al., 2009 [20]	USA	With diabetes mellitus group (White, 36.4%; African American, 46%; Other, 17.5%); Control group (White, 42.3%; African American, 45.3%; Other, 12.4%)	N/A	International Classification of Disease, ninth revision (depressive symptoms)	N/A	<37 week’s gestation)	Prepregnancy or gestational diabetes was independently associated with perinatal depression, including new onset of postpartum depression, in our sample of lowincome new mothers.	Good
Miller et al., 2020 [22]	USA	N/A	N/A	Patient Health Questionnaire—9 items (depression symptoms)	N/A	18–28 week’s gestation)	The diagnosis of GDM was associated with an elevated risk of concomitant pregnancy diagnosis of depression. Given the elevated risk to patients diagnosed with GDM, a more frequent depression screening interval could be considered during the remainder of the pregnancy, such as each prenatal visit.	Good
Pace et al., 2018 [24]	Canada	N/A	(20–44)	International Classification of Disease, ninth revision (depressive symptoms)	N/A	24–28 week’s gestation)	GDM is associated with an increased risk of depression in women particularly during pregnancy highlighting the need to screen for depression and provide supportive interventions during this period.	Good
Rumbold and Crowther, 2002 [26]	Australia	Caucasian, 90%; Asian, 5%; Aboriginal, 1%	29.0 ± 5.0	Edinburgh Postnatal Depression Score—36 items (depressive symptoms)	≥12	Third trimester (36 week’s gestation)	Screening for GDM had an adverse impact on women’s perceptions of their own health.	Poor
Varela et al., 2017 [27]	Greece	Greek, 93.2%; Other, 6.8%	N/A	Edinburgh Postnatal Depression Score—10 items (probably major depression)	≥13	Third trimester	GDM appears to be associated with depressive symptoms in the first week postpartum. Clinical implications and recommendations for future research are discussed, emphasizing the importance of closely monitoring women with GDM who seem more vulnerable to developing depressive symptomatology during the postnatal period.	Poor

Note: N/A, Not available; SD, standard deviation. * The quality of the individual studies was determined using the checklist of Strengthening the Reporting of Observational Studies in Epidemiology (STROBE), the assessment of study quality of included studies by STROBE checklist is shown in Table S2. ^†^ Unit used in enrolment trimester for depression assessment varies across studies, which data were presented either in precise week’s gestation, range of week’s gestation, mean of weeks’ gestation ± SD, or only trimesters.

## Supplementary Materials

The following are available online at https://www.mdpi.com/1660-4601/17/11/3767/s1, Figure S1: Funnel plot of studies evaluating the risk of antepartum depression associated with gestational diabetes mellitus, Figure S2: Funnel plot of studies evaluating the risk of antepartum depression associated with pre-existing diabetes mellitus, Figure S3: Funnel plot of studies evaluating the risk of antepartum depression associated with diabetes in pregnancy, Table S1: Search terms used for final search on 27 December 2019, Table S2: Publication bias was assessed by Egger’s test and Begg’s test for association between GDM and risk of antepartum depression, Table Table S3: Publication bias was assessed by Egger’s test and Begg’s test for association between GDM and risk of antepartum depression. Table S4: Publication bias was assessed by Egger’s test and Begg’s test for association between pre-existing DM and risk of antepartum depression, Table S5: Publication bias was assessed by Egger’s test and Begg’s test for association between diabetes in pregnancy and risk of antepartum depression.

## List of Abbreviations

GDM	Gestational diabetes mellitus
DM	Diabetes mellitus
DIP	Diabetes in pregnancy
RR	Relative risk
CI	Confidence interval
