# Supplementary material for: Diabetes in Pregnancy and Risk of Antepartum Depression: A Systematic Review and Meta-Analysis of Cohort Studies
Source: Int J Environ Res Public Health. 2020 May 26;17(11):3767. doi: 10.3390/ijerph17113767 (PMC7311953; doi:10.3390/ijerph17113767)
Supplement: Supplementary file 1 [file ijerph-17-03767-s001.pdf]

Table S1. Search terms used for final search on 27 December 2019.

Searches	Search terms	Medline via EBSCOHOST	Cinahl via EBSCOHOST	PubMed
#1	mood disorder OR Unipolar depress* OR Depress* OR Depress* disorder OR Major depress* OR Major depress* disorder OR Atypical depress* OR Melancholi* OR Melancholi* depress* OR Melancholi* feature OR Peripartum depress* OR Persistent depress* disorder OR Dysthymic disorder OR Dysthymi*	97,911	6,140	4309
#2	gestational diabetes OR diabetic pregnancy OR diabetes mellitus OR type 1 diabetes mellitus OR type 2 diabetes mellitus OR NIDDM OR Non-insulin dependent diabetes mellitus OR insulin dependent diabetes OR pregnancy diabetes mellitus	135,097	5,360	214188
#3	#1 AND #2	762	66	19

Table S2. Assessment of study quality of included studies by STROBE checklist.

Item.			Included studies									
	**No**	**Recommendation**	**Benute et al., 2010**	**Cripe et al., 2011**	**Daniells et al., 2003**	**Egan et al., 2017**	**Huang et al., 2015**	**Kozhimannil et al., 2009**	**Miller et al., 2020**	**Pace et al., 2018**	Rumbold and Crowther, 2002	Varela et al., 2017
**Title and abstract**	1	(a) Indicate the study's design with a commonly used term in the title or the abstract	0	0	1	1	0	1	1	1	1	1
		(b) Provide in the abstract an informative and balanced summary of what was done and what was found	1	1	1	1	1	1	1	1	1	1
**Introduction**												
Background/rationale	2	Explain the scientific background and rationale for the investigation being reported	1	1	1	1	1	1	1	1	1	1
Objectives	3	State specific objectives, including any prespecified hypotheses	1	1	1	1	1	1	1	1	1	1
**Methods**												
Study design	4	Present key elements of study design early in the paper	0	1	1	1	0	1	1	1	1	1
Setting	5	Describe the setting, locations, and relevant dates, including periods of recruitment,	1	1	1	1	1	1	1	1	1	1

		exposure, follow-up, and data collection										
Participants	6	(*a*) Give the eligibility criteria, and the sources and methods of selection of participants. Describe methods of follow-up	1	1	1	1	1	1	1	1	1	1
		(b) For matched studies, give matching criteria and number of exposed and unexposed	1	0	1	1	1	1	1	1	1	0
Variables	7	Clearly define all outcomes, exposures, predictors, potential confounders, and effect modifiers. Give diagnostic criteria, if applicable	0	1	1	0	1	1	1	1	0	0
Data sources/ measurement	8*	For each variable of interest, give sources of data and details of methods of assessment (measurement). Describe comparability of assessment methods if there is more than one group	1	1	1	1	1	1	1	1	1	1
Bias	9	Describe any efforts to address potential sources of bias	0	0	1	0	1	0	0	0	0	0
Study size	10	Explain how the study size was arrived at	0	1	0	1	0	1	0	0	0	0
	11	Explain how	1	1	1	1	1	1	1	1	1	1

		quantitative variables were handled in the analyses. If applicable, describe which groupings were chosen and why										
Statistical methods	12	(*a*) Describe all statistical methods, including those used to control for confounding	0	1	0	0	1	1	0	1	0	0
		(*b*) Describe any methods used to examine subgroups and interactions	1	1	1	1	1	1	1	1	0	1
		(*c*) Explain how missing data were addressed	0	0	0	0	0	0	0	0	0	0
		(*d*) If applicable, explain how loss to follow-up was addressed	0	0	0	0	0	0	0	0	0	0
		(*e*) Describe any sensitivity analyses	0	0	0	0	0	1	0	1	0	0
**Results**												
Participants	13*	(a) Report numbers of individuals at each stage of study—eg numbers potentially eligible, examined for eligibility, confirmed eligible, included in the study, completing follow-up, and analysed	0	0	1	0	1	1	0	1	1	0

		(b) Give reasons for non-participation at each stage	0	0	1	0	0	1	0	1	0	0
		(c) Consider use of a flow diagram	0	0	0	0	0	0	0	1	1	0
Descriptive data	14*	(a) Give characteristics of study participants (eg demographic, clinical, social) and information on exposures and potential confounders	1	1	1	1	1	1	1	1	1	1
		(b) Indicate number of participants with missing data for each variable of interest	0	0	0	0	0	0	0	0	0	0
		(c) Summarise follow-up time (eg, average and total amount)	0	0	1	0	1	1	0	0	0	0
Outcome data	15*	Report numbers of outcome events or summary measures over time	1	1	1	1	1	1	1	1	1	1
Main results	16	(*a*) Give unadjusted estimates and, if applicable, confounder-adjusted estimates and their precision (eg, 95% confidence interval). Make clear which confounders were adjusted for and why they were included	0	1	0	0	1	1	1	1	0	0
		(*b*) Report category	1	1	1	1	1	1	1	1	0	1

		boundaries when continuous variables were categorized										
		(*c*) If relevant, consider translating estimates of relative risk into absolute risk for a meaningful time period	0	1	0	0	0	0	0	0	0	0
Other analyses	17	Report other analyses done—eg analyses of subgroups and interactions, and sensitivity analyses	0	0	0	1	0	0	0	0	0	0
**Discussion**												
Key results	18	Summarise key results with reference to study objectives	0	1	1	1	1	1	1	1	1	1
Limitations	19	Discuss limitations of the study, taking into account sources of potential bias or imprecision. Discuss both direction and magnitude of any potential bias	1	0	1	1	1	1	1	1	0	1
Interpretation	20	Give a cautious overall interpretation of results considering objectives, limitations, multiplicity of analyses, results from similar studies, and other relevant evidence	1	1	1	1	1	1	1	1	1	1

Generalisability	21	Discuss the generalisability (external validity) of the study results	1	1	1	1	1	1	1	1	1	1
**Other information**												
Funding	22	Give the source of funding and the role of the funders for the present study and, if applicable, for the original study on which the present article is based	0	1	0	1	1	1	1	1	0	1
**Score over 34**			15	21	23	21	23	27	21	26	17	18
**Total score over 22**			10	14	15	14	15	17	14	17	11	12
**Quality**			Poor	Good	Good	Good	Good	Good	Good	Good	Poor	Poor

Table S3: Publication bias was assessed by Egger's test and Begg's test for association between GDM and risk of antepartum depression.

Egger Regression				
	Estimate	SE	CI LL	CI UL
Intercept	0.06	0.41	-0.90	1.02
Slope	0.60	0.10	0.36	0.83
t test	0.15			
p-value	0.882			
**Begg & Mazumdar**				
Δ_{x-y}	-4.00			
Kendall's Tau a	-0.14			
z	-0.49			
p	0.621			

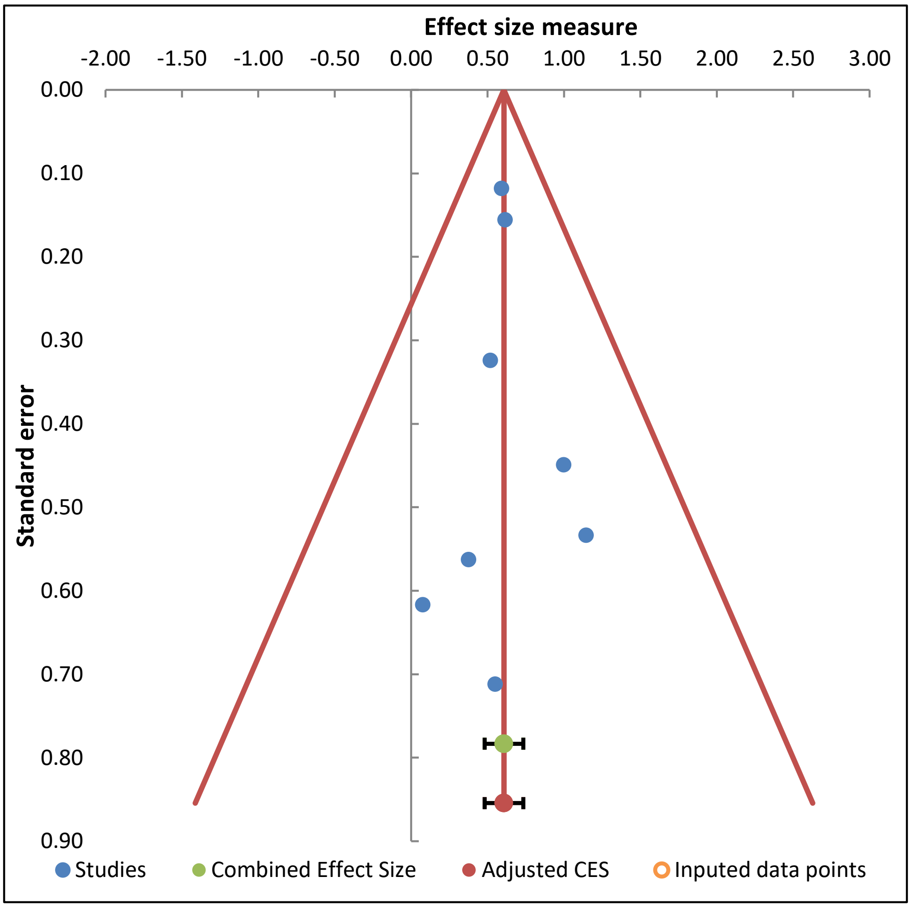

Figure S1: Funnel plot of studies evaluating the risk of antepartum depression associated with gestational diabetes mellitus.

Table S4: Publication bias was assessed by Egger's test and Begg's test for association between pre-existing DM and risk of antepartum depression.

Egger Regression				
	Estimate	SE	CI LL	CI UL
Intercept	-4.44	1.57	-11.19	2.31
Slope	2.70	0.86	-0.99	6.39
t test	-2.83			
p-value	0.216			
**Begg & Mazumdar**				
Δ_{x-y}	-3.00			
Kendall's Tau a	-1.00			
z	-1.57			
p	0.117			

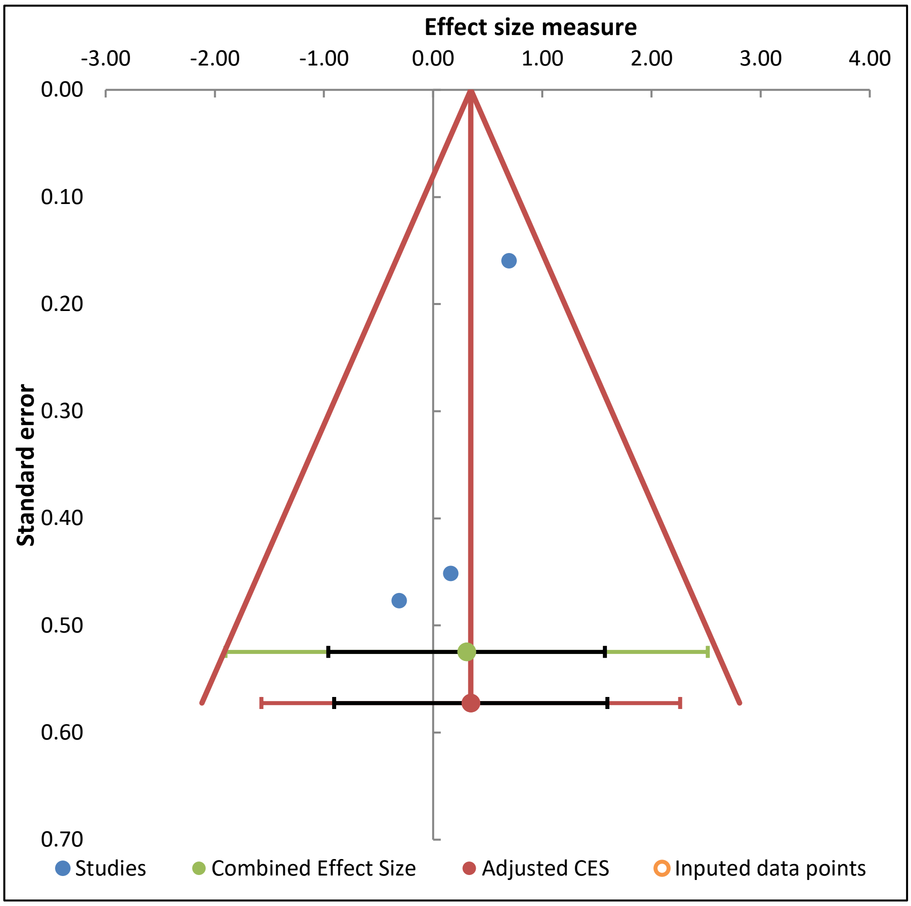

Figure S2: Funnel plot of studies evaluating the risk of antepartum depression associated with pre-existing diabetes mellitus.

Table S5: Publication bias was assessed by Egger's test and Begg's test for association between diabetes in pregnancy and risk of antepartum depression.

Egger Regression				
	Estimate	SE	CI LL	CI UL
Intercept	-0.40	0.51	-1.58	0.78

Slope	0.67	0.11	0.40	0.93
t test	-0.78			
p-value	0.462			
**Begg & Mazumdar**				
Δ_{x-y}	-6.00			
Kendall's Tau a	-0.17			
z	-0.63			
p	0.532			

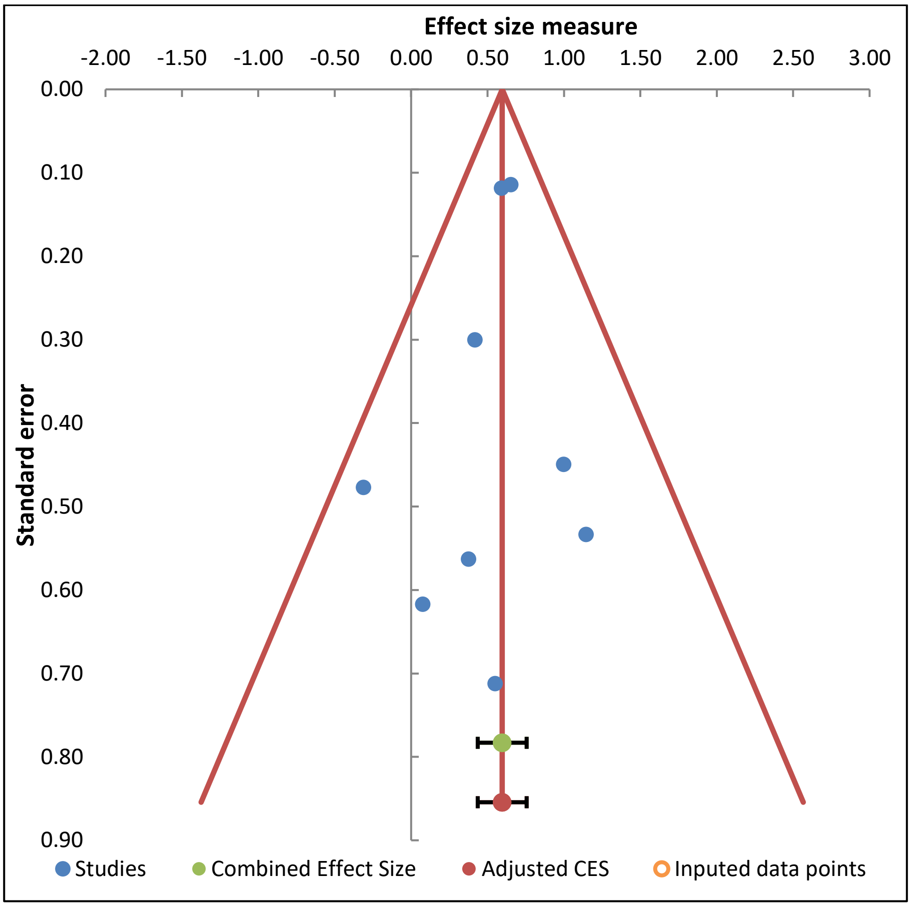

Figure S3: Funnel plot of studies evaluating the risk of antepartum depression associated with diabetes in pregnancy.
